# Artificial Intelligence-Powered Histopathology in Stem Cell Research: Bridging Morphology, Function, and Omics

**DOI:** 10.3390/cimb48090859

**Published:** 2026-08-25

**Authors:** Mashael Saleh Al-Toub

**Affiliations:** Department of Clinical Laboratory Sciences, College of Applied Medical Sciences, King Saud University, Riyadh P.O. Box 60169, Saudi Arabia; altoub@ksu.edu.sa

**Keywords:** artificial intelligence, histopathology, morphology, stem cells, digital pathology, multimodal AI, multi-omics, regenerative medicine

## Abstract

The histopathology area is being redefined with the use of artificial intelligence (AI), providing robust tools to help bridge cellular morphology, functional assays, and multi-omics data in stem cell research. The ability of stem cells to undergo self-renewal and differentiation is key in regenerative medicine, but their research requires the careful characterization of morphological and molecular phenotypic traits. Traditional histopathology is invaluable, but its application can be limited by inter-observer variability and restricted scalability. These limitations are circumvented by AI-based techniques, such as machine learning and deep learning, which are capable of classifying cells, performing quantitative morphometry, and forecasting stem cell behavior. Adding AI to genomics, proteomics, and metabolomics will contribute to the further identification of biomarkers and pathways that regulate stem cell fate. This convergence provides new possibilities for precision medicine, personalized therapies, and translational uses like drug discovery and disease modeling. However, its potential has not yet been realized because of the existing difficulties in data quality, variability, regulatory control, and ethical issues, especially in terms of the transparency and justice of AI systems. Emphasized areas for the future include explainable AI, federated learning, and a multimodal framework that integrates imaging, sequencing, and clinical data. Interdisciplinary partnerships and adequate regulatory frameworks will help AI-enabled histopathology reshape stem cell studies and speed up the process of translating regenerative medicine into clinical applications.

## 1. Introduction

Histopathology has historically been a component of biomedical science, providing essential information about cellular structures and tissue architectures that forms the foundation of disease diagnosis, biomedical research, and therapeutic development [1]. Histopathological analysis has attracted particular attention in stem cell research because morphological features can support the assessment of cellular identity, differentiation, and tissue integration [2]. Traditional histopathological approaches remain valuable, although their interpretation can be affected by subjectivity, inter-observer variability, and labor-intensive manual analysis [3]. These constraints have contributed to the increasing use of computational technologies that can augment human expertise, reduce variability, and accelerate the extraction of meaningful biological information [4].

Artificial intelligence (AI) has emerged as an important computational approach in biomedical research and has increasingly extended the analytical capabilities of histopathology [5]. Machine learning (ML) and deep learning (DL) algorithms can process large image datasets rapidly and reproducibly, supporting automated analysis at a scale that is difficult to achieve through manual assessment alone [6]. In histopathology, AI can identify subtle morphological patterns that may be difficult to recognize consistently by visual examination but may carry biological significance [7]. These developments have established a basis for connecting morphological observations with functional measurements and multi-omics information, providing a more comprehensive framework for investigating stem cell biology [8].

The application of AI to stem cell research has important implications for regenerative medicine. Stem cell properties, including self-renewal and differentiation, are influenced by the surrounding microenvironment and are accompanied by measurable changes in cellular and tissue morphology [9]. Morphological assessment provides valuable phenotypic information, although a comprehensive understanding of stem cell behavior also requires functional and molecular evidence [10]. AI-based analytical frameworks can facilitate the integration of morphological information with genomic, proteomic, and metabolomic biomarkers, supporting the identification of biological signatures, differentiation trajectories, and functionally relevant phenotypes [11].

The importance of the cellular microenvironment is also reflected in recent developments in regenerative biomaterials. Hydrogenated silicene-functionalized scaffolds have demonstrated immunomodulatory bone remodeling and the recruitment of bone marrow mesenchymal stem cells during bone regeneration [12], while bioinspired gradient scaffolds provide spatially organized microenvironments for osteochondral tissue engineering [13]. These examples illustrate how the structural and biochemical properties of engineered environments can influence cellular responses and regenerative outcomes. At a broader biomedical level, multifunctional polyoxometalate nanoreactors further demonstrate the increasing sophistication of engineered platforms that combine structural design with therapeutic functions [14].

AI also provides the scalability required to analyze large datasets generated through high-throughput imaging and multi-omics platforms [15]. This capability is particularly relevant to stem cell research, in which cellular heterogeneity and dynamic transitions during self-renewal and differentiation generate complex multidimensional data. AI can additionally support the translation of stem cell discoveries by facilitating individualized analysis and the development of regenerative strategies informed by patient-specific biological characteristics [16]. The increasing convergence of computational analysis with biomedical engineering is also reflected in emerging nanoscale and data-driven technologies. Nanozyme-integrated nanomotors have been investigated for photothermal-catalytic tumor therapy [17]; defect-engineered metal–organic frameworks have been explored for biomedical applications including drug delivery and tumor therapy [18]; and nanoprobe-based immunoassays provide sensitive strategies for biomarker detection [19]. In parallel, AI-enabled digital biomedical engineering increasingly incorporates computational modeling, medical image analysis, biomaterial design, and drug development within integrated data-driven frameworks [20].

The broader translational landscape also includes emerging nanomedicine platforms with increasingly specialized biological functions. Recent examples include cyanine dye-based nanoparticles developed for combination cancer therapy [21] and fuel-propelled nanomotors designed to enhance targeted drug delivery in acute kidney injury [22]. Although these technologies extend beyond stem cell histopathology itself, they illustrate the growing diversity of biomedical data and engineered systems that require quantitative characterization and integration. Within regenerative medicine, this broader technological evolution reinforces the need for computational approaches capable of relating structural observations to molecular and functional information.

Despite its potential, the introduction of AI into histopathology and stem cell research presents important challenges. Variability in data quality, limited annotated datasets, and algorithmic bias can affect the reliability and external validity of AI-based results [23]. Ethical considerations also remain important, including the use of embryonic stem cells, the handling of patient-derived information, and transparency in AI-supported decision-making [24]. Continued progress in computational methods and data integration therefore needs to develop alongside appropriate validation, standardization, and governance.

In this review, histopathology is used in its conventional morphology-centered sense, referring to the evaluation of cellular and tissue structure, while multi-omics provides complementary molecular information across genomic, transcriptomic, proteomic, and metabolic layers [1,8]. AI is considered an integrative computational framework that connects these distinct data domains for stem cell characterization, fate analysis, and regenerative medicine applications [8,11]. Accordingly, this review examines how AI can bridge morphology, functional assays, and omics technologies to provide a more comprehensive understanding of stem cell biology. Recent developments, current limitations, and future directions are discussed to illustrate how the convergence of computational science, pathology, and stem cell biology may contribute to the development of next-generation regenerative strategies.

## 2. AI-Enabled Computational Analysis in Histopathology

AI contributes to histopathology through defined analytical tasks such as image quality assessment, segmentation, classification, quantitative feature extraction, and prediction [25,26]. In stem cell research, these tasks can support the measurement of subtle morphological states and connect image-derived phenotypes with molecular or functional evidence [27,28,29].

The value of these approaches depends on the biological question, the quality of the imaging data, and the availability of reliable reference labels. For this reason, the following discussion focuses on technologies that are directly relevant to morphology-centered stem cell analysis and computational pathology [26,30].

### 2.1. AI Technologies Relevant to Histopathology and Stem Cell Research

Recent advances extend AI-enabled histopathology beyond task-specific image classification toward self-supervised foundation models, visual language systems, multimodal assistants, and agentic frameworks. These approaches are designed to reuse large-scale learned representations, combine image and text information, or coordinate several analytical steps within a single workflow [31,32,33,34,35].

Recent computational pathology has moved toward self-supervised and foundation models that are pre-trained on very large collections of histology images and then adapted to different downstream tasks. The UNI foundation model was pre-trained on more than 100 million image patches from over 100,000 whole-slide images and was evaluated across a broad range of computational pathology tasks, while the CONCH visual language model combined histopathology images with biomedical text to support classification, segmentation, captioning, and image–text retrieval [31,32].

Natural language processing (NLP) and large language models (LLMs) extend AI beyond image analysis by processing pathology reports, laboratory records, and biomedical text. PathChat demonstrated how visual and natural language information can be combined in an interactive pathology assistant, while agent-based LLM systems have also been developed to extract and structure information from pathology reports for downstream analysis [33,36].

Multimodal AI provides a direct computational route for integrating histological morphology with genomic, transcriptomic, proteomic, clinical, and textual information. Such models can relate visible phenotypes to complementary biological signals and support the more complete interpretation of tissue and cellular states [33,37].

Intelligent agent systems represent a further emerging direction in pathology. Agentic models can perform sequential evidence-seeking steps, coordinate image analysis with language-based reasoning, and revisit regions of interest when additional evidence is required. Recent systems such as PathFound and Pathology-CoT illustrate this shift toward multistep and human-interpretable pathological reasoning, although these approaches remain at an early stage of validation [34,35].

**Figure 1:**
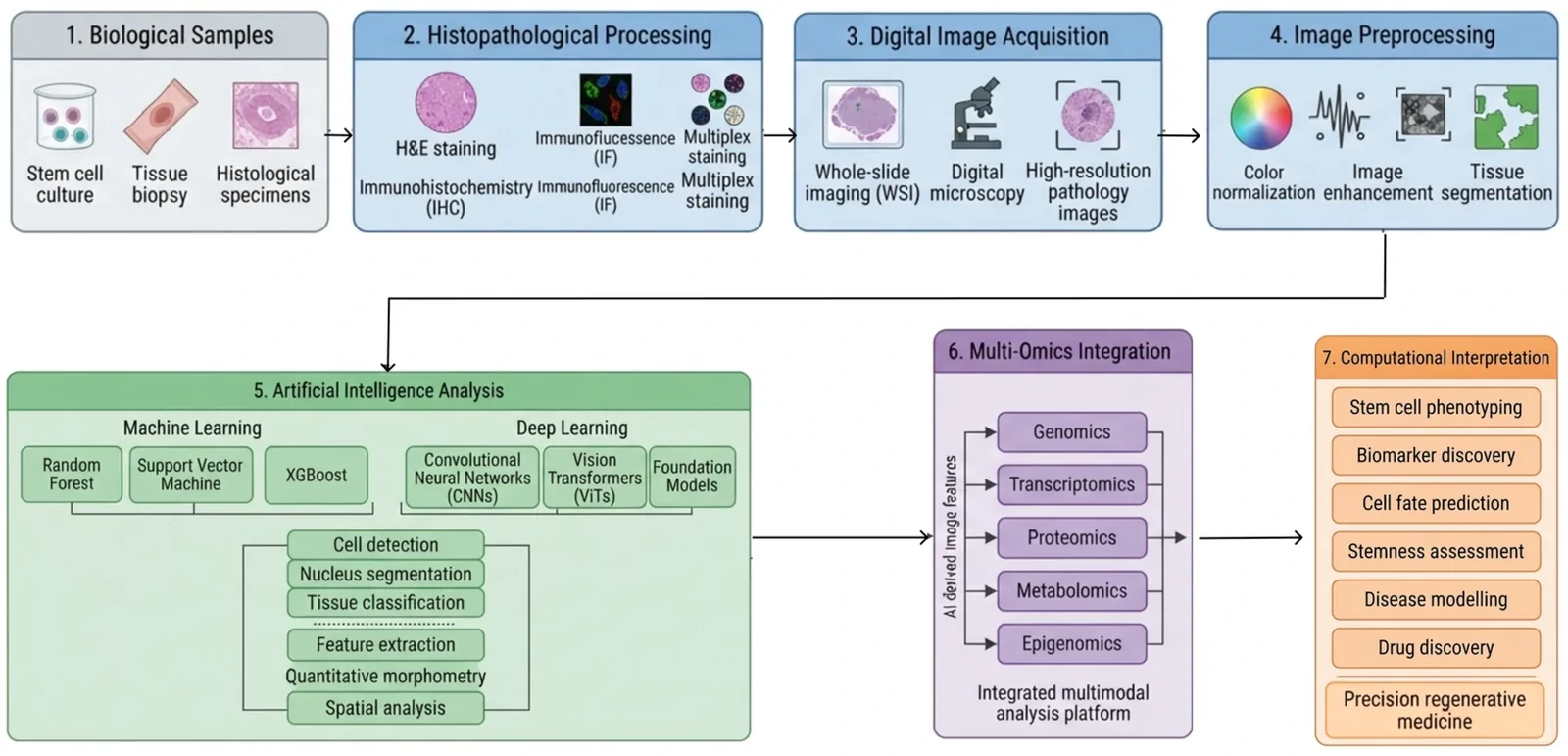
Artificial intelligence workflow for stem cell histopathology, illustrating the progression from biological sample acquisition and digital pathology to image preprocessing, AI-based analysis, multi-omics integration, and computational interpretation for stem cell phenotyping and precision regenerative medicine (created by the author).

The integrated workflow is summarized in Figure 1, which positions histopathological imaging as the morphology-centered data source and connects it with complementary molecular information through AI-based analysis [31,37].

### 2.2. Machine Learning Algorithms in Pathology

AI algorithms are diverse and sophisticated, which explains why artificial intelligence will be a transformative technology in histopathology [38]. The different classes of algorithms have their own unique advantages, which allow meaningful biological information to be extracted from complex histological data. The judicious use of machine learning has opened up potential capabilities in classification, segmentation, and predictive modeling in stem cell research, in which morphology and microenvironmental context are important predictors of functionality [39].

The earliest algorithms to be applied to histopathology included classical algorithms like support vector machines (SVMs), decision trees, and random forests [40]. SVMs are well adapted to distinguish fine changes between a pluripotent cell and a differentiated cell, such as nuclear or cytoplasmic morphology variations [27,28]. Random forests and decision trees provide interpretable models with high reproducibility that can be used to classify histological patterns into discrete stages (e.g., early versus late differentiation). Their interpretability is a strength for the translational environment, where clear rules for decision-making are appreciated [41]. Although the classical models are not as powerful as deep neural networks, they can still be used in situations where the data size is small, there are few annotations, or computer resources are limited.

Artificial neural networks (ANNs) and specialized architectures, mainly Convolutional Neural Networks (CNNs), have made the most important contributions to computational pathology. CNN-based networks are useful in image classification and segmentation [42], allowing high-resolution tissue architecture analysis. For instance, CNNs have been utilized to outline stem cell colonies in phase-contrast images, spatial niches in organoid cultures, and stromal and epithelial compartments in regenerative tissue models [43]. Architectures like U-Net can be segmented down to the pixel level, enabling the creation of detailed cellular boundary maps that can be used to drive quantitative morphometric analyses [44]. Meanwhile, more intricate architectures, such as ResNet, have allowed the correct detection of fine histological information, such as the emergence of apoptosis or abnormal differentiation.

The use of transfer learning based on pre-trained models on large datasets such as ImageNet and fine-tuning on small datasets based on histopathology-specific data has been a significant innovation [45]. However, this method exhibits one of the most important issues in stem cell pathology: the limited number of annotated images. Transfer learning enables the rapid creation of strong models and eliminates the large labeling requirements due to the use of generalized visual features based on massive data [46]. Transfer learning has been especially valuable in the difficult task of detecting rare events, like finding rare subpopulations of stem cells or determining the existence of subtle morphological features, which is an early sign of malignant transformation [47].

Recent efforts have also focused on machine learning to detect rare events, as well as predictive modeling. Early detection of abnormal differentiation and/or genomic instability is important in terms of therapeutic safety. Algorithms that have been trained on high-resolution histological data can be used to identify rare malignant clones or early fibrotic alterations [48,49]; this can be used as an additional quality control step prior to clinical application. Moreover, dynamic processes of differentiation can be followed with the help of predictive models based on incorporating temporal histological data, which provide information about lineage commitment and plasticity [50].

## 3. Stem Cell Biology

Stem cells are the cornerstone of regenerative therapy, and they are still the focus of study in developmental biology, tissue repair, and disease modeling. Stem cells are characterized by their two distinct abilities to self-renew and differentiate and can therefore be used to model cell fate choices, as well as to repair or replace damaged tissues therapeutically [51]. The histopathological area has its own challenges and opportunities when applied in the context of stem cell biology [52]. Morphological analysis has long been essential in the ability to define the states of stem cells [53]. The combination of histopathology and multi-omics data with artificial intelligence therefore offers a more comprehensive means of interrogating stem cell biology.

### 3.1. AI-Enabled Characterization of Stem Cell Systems

AI-enabled stem cell research focuses on measurable phenotypic states and their relationships with self-renewal, differentiation, and functional behavior. Quantitative imaging can capture the colony architecture, nuclear features, cell shape, spatial organization, and temporal changes, providing non-destructive phenotypic information that can be analyzed together with molecular and functional assays [27,28,29].

In embryonic (ESCs) and induced pluripotent stem cell (iPSCs) cultures, computational morphology can support pluripotency assessment and quality control by detecting changes in colony structure and cellular appearance. Machine learning and deep learning approaches have been used to distinguish favorable and unfavorable colony phenotypes and to improve the consistency of image-based assessment across large culture collections [28,29,54,55].

For adult stem and progenitor cells, AI can assist the study of lineage commitment by combining morphological measurements with longitudinal imaging and functional information. Deep learning analysis of hematopoietic progenitors has shown that future lineage choices can be predicted from cellular morphology and movement before conventional lineage markers become evident, while advanced microscopy provides complementary structural information during mesenchymal stem cell differentiation [56,57,58].

Organoid systems provide a further application because their three-dimensional organization produces spatially heterogeneous and dynamic phenotypes. AI-based image analysis can support segmentation, quality assessment, and developmental trajectory prediction in organoids, including the early prediction of later structural outcomes from time-lapse imaging [43,59,60].

Cancer stem cell research also benefits from computational phenotyping because these populations can be difficult to distinguish by morphology alone. Image-based analysis combined with molecular profiling can support the investigation of phenotypic heterogeneity, treatment resistance, and stem-like tumor states [49,61].

A key future direction is the integration of longitudinal imaging with multi-omics and functional readouts so that image-derived predictions can be linked to molecular programs and validated biological outcomes. This approach is particularly relevant to fate prediction, where early morphological information may provide predictive signals before terminal differentiation is established [37,57,60,62].

### 3.2. Stem Cell Differentiation

Stem cell differentiation represents one of the most fundamental processes in biology, wherein pluripotent or multipotent cells gradually commit to specialized fates [63]. This transformation is orchestrated by tightly regulated networks of transcriptional programs, epigenetic modifications, and environmental signals within the cellular niche. From a histopathological perspective, differentiation is reflected in gradual yet discernible changes in cell morphology, nuclear architecture, cytoplasmic composition, and tissue organization [64]. These phenotypic transitions, although sometimes subtle, provide important clues about lineage commitment and functional maturation. In traditional settings, differentiation is assessed through a combination of morphological observation and lineage-specific staining [65]. However, such methods are constrained by subjectivity, limited throughput, and reliance on predefined markers. The emergence of AI-driven image analysis offers a powerful alternative by enabling the unbiased detection of differentiation-associated features that may not be apparent to human observers [27,66].

Recent experimental studies further illustrate the diversity of differentiation pathways relevant to regenerative medicine. Annexin A1 (ANXA1) has been shown to promote the odontogenic differentiation of dental pulp stem cells through the activation of the ERK/mitogen-activated protein kinase signaling pathway, supporting its relevance to dental tissue regeneration [67]. In bone regeneration, PBVHx nanoparticle-mediated ginsenoside Rg3 nanocarriers enhanced the osteogenic differentiation of stem cells, linking biomaterial-mediated regulation with osteogenic lineage commitment [68].

Stem cells experience typical variations in size, shape, and organization in the course of differentiation, which can be documented by the use of histopathological visuals [56]. An example lies in the fact that neuronal development usually results in long cells with extended neurites, whereas cardiomyocytic development is characterized by striated fibers and organized contractile forms [69]. Conventionally, such observations are confirmed by immunohistochemical markers like βIII-tubulin in neurons or troponin in cardiomyocytes. However, these markers can only establish differentiation once it has been established, while computational methods can be used to anticipate lineage paths earlier by finding morphological precursors [57]. CNNs, for example, have been trained to identify early visual features of mesodermal and endodermal differentiation with high concordance with transcriptomic data [58]. These prognostic features broaden the horizons of histopathology from descriptive evaluation to proactive modeling.

Another complication in the stem cell differentiation process is the phenomenon of plasticity. Stem cells are also capable of adopting alternate or transitional states that do not follow the canonical differentiation programs [70]. Dedifferentiation, in which committed cells differentiate back into progenitor-like phenotypes, is one of these challenges and frequently takes the form of ambiguous morphologies that obscure conventional histopathological lines [71]. In these cases, unsupervised machine learning algorithms have been of great value in clustering cells according to their subtle morphological embeddings, thus determining intermediate states that were otherwise unknown [72]. Such power to chart the cell-state space is especially useful in studies of developmental plasticity and in enhancing the fidelity of differentiation efforts.

Another important point of intersection between histopathology and AI is pathological differentiation. In regenerative medicine, any incomplete or misdirected differentiation may cause safety hazards, such as teratoma formation due to remaining pluripotent cells or the production of fibrotic tissue rather than functional parenchyma [73]. The initial signs of these negative outcomes in histology are usually very mild and cannot be detected easily by manual analysis. By contrast, AI-based predictive models can identify patterns that precede overt pathology, flagging colonies or tissues at risk of abnormal differentiation [74]. This application is particularly relevant for clinical translation, where rigorous quality control is required to ensure the safety of stem cell-derived therapies. Moreover, in organoid systems, where differentiation must proceed in a spatially coordinated manner, computational models can detect imbalances between lineages that may compromise organ-level functionality [75].

Linking morphology with functional outcomes remains a central challenge in stem cell biology. Functional assays such as electrophysiological recordings or metabolic profiling provide definitive readouts of differentiation but are often destructive, resource-intensive, and of low throughput [10,29]. AI-enhanced histopathology offers an attractive alternative by correlating morphological signatures with functional states. For instance, cardiomyocyte contractility has been correlated with sarcomere alignment, a feature that can be automatically quantified from histological images [76]. Similarly, neuronal network activity has been linked to morphological parameters such as neurite density and branching complexity, both of which can be detected computationally [77]. Such correlations enable the non-destructive, high-throughput inference of functionality from morphology, accelerating the evaluation of differentiation protocols.

## 4. Histopathological Techniques

In research and clinical diagnosis, tissue analysis is based on histopathological methods. In the case of stem cell biology, these methods offer an invaluable means of assessing the morphology of cells, their lineage capacity, and tissue integration [12]. In contrast to a purely molecular approach, histopathology enables the visualization of the cellular and structural nature directly, thus connecting microscopic images to a functional and developmental process. Traditional techniques such as fixation, staining, and light microscopy have given way to new technologies such as immunolabeling, advanced microscopy, and digital pathology, which have increased resolutions and analysis capabilities [78]. With the growing convergence of stem cell research, AI, and multi-omics, histopathological methods are now being reconsidered, yielding not only descriptive data [79] but also high-content data that can be used in computational models. It is critical to understand the continuum of methodological techniques, beginning with sample preparation and concluding with high-end imaging, in order to place the use of AI-driven histopathology within the context of the improvement of stem cell research and regenerative medicine [80].

### 4.1. Tissue Preparation and Staining

Histopathological evaluation starts with tissue preparation, which is an essential stage that should preserve the structural and molecular integrity of the sample [81]. Fixation is performed to fix cellular proteins and inhibit autolysis, so that the morphology of the stem cell-derived tissues and organoids is not changed [82]. After fixation, the mechanical stability of the section is ensured by embedding it in paraffin or cryoprotective media; this results in thin sections that can be examined under a microscope [83]. Although these are technical steps, they play an important role in image quality and downstream analysis. For instance, morphological artefacts may occur due to poor fixation, whereas an overabundance of cross-linkages can conceal antigens, and further immunohistochemistry (IHC) will be hampered by cross-linkages.

The hematoxylin and eosin (H&E) staining method is fundamental in histopathology and provides a general view of the tissue structure. The nuclei are stained with hematoxylin in deep blue or purple, whereas the cytoplasm and extracellular structures are stained with eosin in pink, clarifying the distribution of cells and their organization in tissues [84]. H&E is frequently employed in the initial screening in stem cell studies to assess the differentiation of tissue grafts, engineered constructs, or organoids [85]. H&E indicators can thus be used to determine the presence of glandular structures, the layering of neurons, or striated muscle fibers before more specific molecular analyses are performed. Although H&E is essential in evaluation, it lacks molecular specificity, which requires the use of a supplementary staining method.

The use of IHC gives this specificity by allowing one to visualize protein expression in vivo. IHC can be used to identify pluripotency lineage markers (OCT4, SOX2, or NANOG) or lineage commitment markers [86]. IHC finds specific application in stem cell research, where it is used to validate differentiation protocols, as well as to determine heterogeneity within populations. Furthermore, IHC based on multiplexing allows the detection of multiple markers in real time, providing information about cell states and cellular interactions in complicated microenvironments [87].

In addition to the traditional IHC, more complex labeling methods have been developed to deal with the complexity of stem cell systems. The visualization of mRNA transcripts can be achieved using fluorescent in situ hybridization (FISH) or its high-throughput derivatives, which connect the morphological context with gene expression [88]. Barcoded antibody arrays with either imaging mass cytometry or multiplex immunofluorescence increase the dimensionality of tissue profiling, measuring dozens of markers in a single sample [89]. These innovations do not only increase the granularity of histopathological analysis but create high-dimensional data that are ready to be combined with machine learning methods. Tissue preparation and staining methods help to maintain structural and molecular data, which form the initial input on which AI-guided histopathology is based.

### 4.2. Microscopic Imaging Techniques

Microscopic imaging is the interpretative basis on which prepared tissues are observed. The most commonly used modality is classical light microscopy, which is a rapid and cost-effective visualization technique for stained samples [90]. It is applicable in routine differentiation studies, the structural organization of engineered tissues, and the detection of pathological changes such as necrosis or fibrosis [91]. The use of two-dimensional images, however, restricts the ability to examine spatial complexity, which is present in stem cell-derived organoids and tissues.

One of the solutions to these limitations is fluorescence microscopy, which allows one to visualize certain proteins or structures that have been labeled with a fluorescent marker [92]. This method is commonly used in stem cell research, where the dynamic measurement of differentiation markers, cytoskeleton structures, and cell–cell interactions can be achieved. Notably, recent advances in spectral unmixing and multiplexed fluorescence imaging mean that they can now be used to observe many targets simultaneously, making it possible to gain systems-level information about lineage allocation and microenvironmental signals [93].

Confocal and multiphoton microscopy has become essential in achieving high-resolution and three-dimensional visualization. Confocal microscopy is a method based on point illumination and optical sectioning to remove out-of-focus light and create sharp images of the cellular structure in thick tissues [94]. Multiphoton microscopy also increases the penetration depth to allow the imaging of intact organoids and in vivo grafts with less phototoxicity [95]. Such modalities have been very useful in mapping differentiation gradients and localized signaling in tissues generated by stem cells and provide information that is unavailable with two-dimensional techniques.

Electron microscopy (EM) is a technique that enables a detailed description of the structure on the nanometer scale—that is, the visualization of subcellular structures, e.g., mitochondria, the endoplasmic reticulum, and synaptic contacts [96]. EM has been applied to stem cell studies to verify the maturation of cardiomyocytes by determining the presence of sarcomeric structures or to measure the presence of synaptic connections in neuronal cultures [97]. Despite requiring considerable resources, EM cannot be surpassed in terms of the unquestionable structural validation of differentiation results.

**Figure 2:**
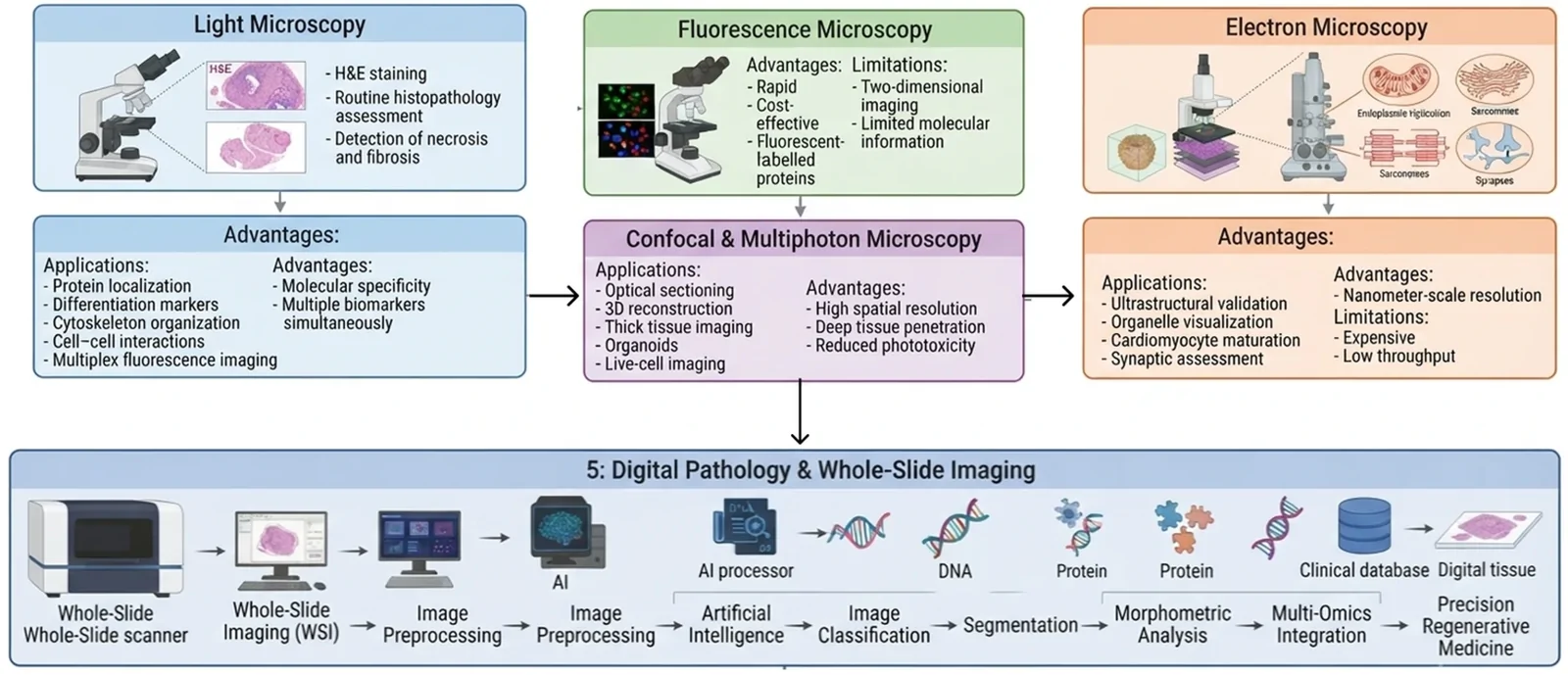
Microscopy foundations of stem cell histopathology: from conventional visualization to AI-compatible digital imaging platforms. A diagram of the evolution of imaging technology for stem cell identification, from conventional microscopy to advanced imaging methods and computational pathology. Light microscopes can routinely evaluate tissue architectures and pathological changes, while fluorescence microscopes provide a molecular level of visualization of differential markers, protein locations, and cell interactions. Confocal and multiphoton microscopes offer high-resolution three-dimensional images of thick tissues and organs, while electron microscopes provide ultra-structural validation at the nanometer scale. Digital pathology and comprehensive imaging extend microscopy to the computational framework, allowing image preparation, AI-driven classification, segmentation, and quantitative morphological analysis. The integration of image data with multidisciplinary approaches facilitates the complete differentiation of stem cells and supports precise applications of regenerative medicine (created by the author).

Recently, technological innovations in digital pathology and whole-slide imaging (WSI) have made microscopy a computationally feasible field. WSI enables the sharing of complete tissue sections at a high resolution and with strong reproducibility, and it enables large-scale quantitative analysis remotely [98]. Digital slides also offer a platform on which to train and validate AI algorithms to be used in automated classification, morphometric analysis, and predictive modeling in stem cell research [99]. Combining the capabilities of imaging data with genomic and clinical data also brings digital pathology to new heights as a core technology in precision regenerative medicine. Figure 2 summarizes the major microscopy techniques used in stem cell pathology and shows how advances in imaging technology have facilitated the transition from conventional microscopy to digital pathology and AI-assisted image analysis.

## 5. Integrating Omics Data

Histopathology is the classical means of obtaining morphological insights into stem cell biology, but the complexity of differentiation, lineage plasticity, and disease modeling necessitates more than just structural insight [100]. The inclusion of omics technologies-genomics, transcriptomics, proteomics, and metabolomics-introduces a molecular aspect, bridging the connection between cellular structures and regulatory pathways, signal transduction, and metabolic conditions [101]. This unification is especially important during the age of AI, when the multimodality of data inputs allows the more accurate prediction of stem cell fates, the earlier identification of aberrant differentiation, and the identification of therapeutic targets [102].

Multi-omics approaches in regenerative medicine give a systems-level view to determine the safety, efficacy, and reproducibility of products of stem cells [103]. The genomic profiling of iPSCs can detect subclonal mutations, whereas proteomic and metabolomic profiling can detect concealed heterogeneity in a population otherwise regarded as uniform [104]. When these datasets are compared with histopathological data, the resulting models not only confirm cellular phenotypes but also give mechanistic information about differentiation dynamics. AI integration further improves this process because it can identify patterns in large, heterogeneous datasets, connecting morphology to multi-omics to confirm results at the levels of single cells and tissues [105].

Therefore, the combination of histopathology, omics, and AI is a revolutionary paradigm in stem cell biology. Genomics and transcriptomics provide a blueprint for cell potential, and proteomics and metabolomics enable the functional capture and contextualization of these signals via histopathology in the context of the tissue architecture [106].

### 5.1. Genomics in Stem Cell Research

Genomic analysis has emerged as the foundation of stem cell biology, providing in-depth information about the regulatory processes that control pluripotency, differentiation, and lineage specification [107]. Whole-genome sequencing (WGS) and whole-exome sequencing (WES) have already been widely used to identify genetic variants in ESCs and iPSCs [108]. These approaches offer key insights into the stability of these genetic variants, which is a major factor in the safety of stem cell-based therapies. In addition to bulk sequencing, single-cell genomics has become a revolutionary technology that clarifies the issue of cellular heterogeneity through the acquisition of genomic variations on an individual cell scale [109]. This specifically applies to the case of stem cell populations, in which small subclones that contain deleterious mutations would otherwise not be detected by bulk assays.

The combination of genomic information with histopathology and morphology is anticipated as scientists endeavor to bring genetic data within a structural context. For instance, genetic defects affecting genes that regulate cytoskeleton assembly or extracellular matrix remodeling may take the form of minor morphological defects that can be observed in histological sections [110]. Multimodal models based on AI are becoming able to connect such genomic changes with image-based characteristics, which can be used to predict the differentiation course and disease predisposition [111]. Single-cell genomic profiling analysis coupled with histological imaging in neural differentiation studies has shown specific lineage bifurcations controlled by transcription factor networks [112], offering a mechanistic understanding of the morphological heterogeneity of neural progenitors.

Genomic instability in iPSCs can predispose patients to tumorigenesis, which can be observed histologically by teratoma assays [113]. Through the correlation of genetic changes and the morphological features of pathological differentiation, AI-directed analyses are capable of determining warning signals at an early stage, which guarantees increased safety in the field of clinical application. Likewise, in disease modeling, patient-derived iPSCs can be used together with genomic sequencing and histological analysis to enable the recreation of disease-specific phenotypes, e.g., cardiomyopathies or neurodegenerative pathologies, in vitro [114].

This association between morphology and genomics is further improved with the emergence of integrative methods like spatial transcriptomics [115]. These methods can be used to map the pattern of gene expression directly onto histological tissue structure, preserving the spatial context, to show how genomic drivers coordinate cell–cell interactions and tissue organization. Spatial transcriptomics has been applied to map developmental gradients and lineage zoning in organoid systems, which can be directly compared to histological sections [116]. The significance of AI aligns with the huge volumes of spatial transcriptomics data and digital histopathology, making it possible to align these data and cross-modally interpret them automatically.

Genomics offers a molecular basis for stem cell fate choices, and its interpretative ability is optimized when combined with the histopathological background [117]. Integrative models combine genetic data with morphological and architectural characteristics to develop a holistic model, which is essential in both basic discovery and translational applications in regenerative medicine.

### 5.2. Proteomics and Metabolomics

While genomics determines the potential of cells, proteomics and metabolomics encode the functional states that trigger differentiation and cellular behavior. Proteomics, which is mostly founded on mass spectrometry (MS) technology, provides a detailed report of the protein abundance, post-translational alterations, and signaling pathways operating in the stem cell mass [118]. These proteomics data give direct evidence of the functioning machinery, showing how differentiation pathways are implemented at the molecular scale. Recent techniques like tandem mass tag (TMT) labeling, data-independent acquisition (DIA), and single-cell proteomics enable increased depths and resolutions in stem cell characterization [119].

Proteomics has also been of great importance in clarifying the signaling pathways involved in stem cell fate. Phosphoproteomic profiling has revealed kinase-mediated cascades that govern the switch between pluripotency and lineage commitment [120]. Such datasets, when combined with histopathological data, show the nature of manifestations of changes in signaling in morphological phenotypes. Computational frameworks based on AI take this integration one step further by revealing the proteomic signatures of the following histological outcomes: nuclear-to-cytoplasmic ratios, cytoskeletal organization, and extracellular matrix deposition [121]. This type of understanding can be used to predict the states of stem cells without using destructive techniques.

Metabolomics is an adjunct to proteomics in that it profiles the small-molecule intermediates of cellular metabolism, providing a snapshot of the bioenergetic and biosynthetic environment [122]. Metabolic reprogramming is closely linked to stem cell differentiation, with pluripotent cells preferring glycolysis and differentiated cell lines progressively depending on oxidative phosphorylation [122]. Stage-specific metabolic changes that can be attributed to morphological differentiation hallmarks have been identified using high-resolution metabolomics and nuclear magnetic resonance (NMR) or liquid chromatography–mass spectrometry (LC-MS) [123]. For instance, neuronal differentiation is commonly linked to augmented lipid metabolism, whereas cardiomyocyte maturation is allied with augmented mitochondrial activity. Such metabolic properties are measurable and can be compared to histological indications of differentiation, giving an integrative view of structure and function [124].

In particular, AI-based models can effectively combine metabolomics data and morphology. For example, CNNs trained on histological images of differentiating cardiomyocytes have been coupled with metabolomics profiles to predict contractile capacity [125]. Similarly, in neural stem cell systems, oxidative stress metabolomics states have been correlated with histopathological indications of abnormal differentiation, allowing the early detection of dysfunctional pathways [126]. These strategies show how morphology, proteomics, and metabolomics could be integrated into predictive models that have the potential to guide basic biology as well as therapeutic use.

The combination of proteomics and metabolomics data with histopathology is not an easy task. High dimensionality, sample preparation variability, and low spatial resolutions are still limitations to routine use. However, new methods in spatial proteomics and metabolomics now enable one to map the distributions of proteins and metabolites in tissue sections [127]. These spatially resolved data, which are compatible with histological data, provide previously unidentified information about the distribution of functional states in cellular niches. These data, together with machine learning, form a multilayered view of tissue organization that surpasses the ability of morphology to describe the structure of tissues.

## 6. Morphological Analysis in Stem Cell Research

Morphology remains one of the most accessible and non-destructive readouts in stem cell research. The nuclear-to-cytoplasmic ratio, colony architecture, cell polarity, cytoskeletal organization, spatial density, and tissue patterning can change during self-renewal, differentiation, stress, or pathological transformation [29,128].

Digital pathology and computational morphometry extend these observations by converting visual phenotypes into reproducible quantitative measurements that can be related to molecular and functional data [63,64].

### 6.1. Quantitative Morphometry

Quantitative morphometry converts visual assessments into objective measurements of cellular and tissue properties. Features such as cell size, shape, aspect ratio, perimeter complexity, colony organization, and nuclear architecture can be extracted reproducibly from microscopy images and used to characterize phenotypic transitions in stem cell populations [56,57,129].

Morphometric measurements can also be associated with differentiation potential and lineage commitment. Combining cellular and colony-level features is particularly informative because the stem cell phenotype is expressed across multiple spatial scales, from individual cell structures to colony organization [130].

A major benefit of quantitative morphology is its ability to connect non-destructive imaging with molecular readouts. Morphometric features can be analyzed together with chromatin, proteomics, and other omics measurements to identify relationships between visible phenotypes and underlying cell states [11,23].

### 6.2. Morphological Features of Stem Cells

Morphological features can be useful as diagnostic indicators of the state of a stem cell, offering insight into its functional capabilities. A small and round form with a high nuclear-to-cytoplasmic ratio, large nucleoli, and compact colony formations are characteristic features of undifferentiated stem cells, especially ESCs and iPSCs [25]. These characteristics reflect their proliferative and transcriptionally active condition. With further differentiation, cells develop lineage-specific morphologies: neurons extend neurites and form network-like structures, cardiomyocytes organize themselves into striated fibers with spontaneous contractions, and epithelial cells develop polarized layers with distinct junctions [62]. These morphological characteristics are visual markers of lineage faithfulness and functional maturation.

Morphology is essential in the quality control of stem cell cultures by differentiating between non-differentiation and differentiation [54,55]. The undifferentiated remaining cells may be hazardous, such as in the formation of a teratoma when it is transplanted into the body. A large number of these cells can be detected even when they are scarce, and, with the support of AI, the safety of therapeutic interventions based on stem cells is guaranteed [55,73]. Smart models have demonstrated high accuracy in identifying small populations of undifferentiated pluripotent stem cells (PSCs) in differentiated cultures and are more sensitive than conventional staining techniques [55].

Besides normal differentiation, morphological analysis is also useful in identifying rare or abnormal cell populations. Genomic instability or pathological transformation may be indicated by an aberrant nuclear morphology, colony architecture, or cytoplasmic structure [48,113]. Patient-derived iPSCs tend to reproduce morphological defects with a particular form of disorder. Neurons derived from the iPSCs of patients with neurodegenerative diseases have shorter length and branching neurites, whereas cardiomyocytes from patients with dilated cardiomyopathy have disorganized sarcomeres. These disease-specific morphologies can offer not only mechanistic information but also phenotypic endpoints in therapeutic testing [48].

Morphological analysis based on AI is especially useful in disease modeling. Subtle deviations that can be missed by a human observer can be detected by automated systems, and the stratification of disease subtypes and prediction of progression are possible [131]. Morphological changes in stem cell-derived organoids have been associated with mutational signatures determined by genomics in oncology research, providing a potent platform on which to realize precision oncology. In metabolic diseases, AI has been used to associate the morphological characteristics of hepatocyte-like cells with metabolomic patterns, revealing a connection between structure and function.

## 7. AI Applications in Stem Cell Histopathology

AI applications in stem cell histopathology can be grouped according to the biological decisions that they support, including culture quality control, phenotypic classification, fate prediction, disease modeling, and the assessment of therapeutic or drug responses [131,132,133,134].

In stem cell histopathology, AI can be used in two main applications, which consist of automated cell classification and the predictive modeling of stem cell behavior. Automated classification addresses the requirement for the scalable and standardized identification of stem cell subtypes, which should be consistent across laboratories and in the clinical environment [10]. Meanwhile, predictive modeling uses temporal and multi-omics data to predict the differentiation direction and therapeutic outcome, eliminating the gap between in vitro results and clinical practice.

### 7.1. Automated Cell Classification

Automated classification provides a consistent approach to distinguishing phenotypic states in large image collections. In stem cell systems, it can support the identification of undifferentiated colonies, differentiated progeny, abnormal growth patterns, and residual pluripotent cells that may be relevant to culture quality and therapeutic safety [85,132].

The clinically relevant advantage is scalability and reproducibility. Automated systems can screen large image collections, flag atypical regions, and support the expert review of ambiguous or safety-relevant cases. Performance should be evaluated with task-appropriate measures and independent validation, particularly when rare abnormal states are the target [85].

In regenerative medicine and drug discovery, image-based classification can support the quality control of differentiated cultures and high-throughput phenotypic screening by providing standardized measurements across repeated experiments [55,134].

The other positive feature of automated classification is its flexibility. Transfer learning enables models trained on large image data (such as ImageNet) to be fine-tuned on small domain-specific data for particular stem cell histopathology tasks [45,46]. This is flexible and can hasten development and increase applicability, even in laboratories with fewer annotated images. To further enhance explainable AI (XAI) techniques, saliency maps and Grad-CAM are being incorporated more often to demonstrate the regions of the image that contribute to the classification [41,133]. This interpretability is key to the development of trust between researchers and clinicians and in ensuring that AI models complement human expertise instead of eliminating it.

### 7.2. Predictive Modeling of Stem Cell Behavior

In addition to classification, AI can be used to predict or model stem cell behavior and provide a dynamic model of cellular processes. The differentiation of stem cells, lineage specification, and functional maturation is a complicated process that is affected by different molecular, environmental, and temporal signals [9,50]. Conventional approaches are based on retrospective studies, which only record endpoints and do not predict patterns. AI overcomes this weakness by combining longitudinal data from time-lapse microscopy, transcriptomics, proteomics, and metabolomics, producing models that can simulate and predict stem cell fates [131].

DL and time-lapse imaging serve as an excellent basis for predictive modeling. Morphological dynamics that predict differentiation can be learned by algorithms trained on sequential image datasets, and earlier differentiation can be predicted by the algorithm before traditional markers have been developed [57]. For example, an increase or decrease in cell motility or nuclear shape may be an indication of the transition to neural or mesodermal lineages. These prediction capabilities make it possible to make decisions beforehand in culture systems to minimize waste and enhance the yields of desirable cell types. This is further enhanced by integration with omics datasets to enhance predictive modeling. Multimodal inputs such as genomic variants, transcriptional signatures, and proteomic states can be integrated into AI frameworks, along with morphological data, and they generate comprehensive descriptions of stem cell behavior [37]. For example, neural architectures using recurrent neural networks (RNNs) and graphs have been applied to predict differentiation programs, and autoencoders have been utilized to reduce the dimensionality of complex data without signal detachment [37,39]. These methods allow the individual modeling of therapeutic responses that are connected with patient-specific cell morphologies generated by iPSCs and omics-informed predictions of functionality [38].

Predictive models would also be useful in regenerative medicine to predict therapeutic efficacy and safety prior to transplantation and reduce the risks of tumorigenicity or incomplete differentiation. AI also finds application in drug discovery, in predicting the responses of stem cells to pharmacological treatment with virtual screening and the elimination of expensive and time-intensive assays [48]. These models can also be used to stratify patients, as iPSCs from different patients may respond differently to the same treatment. Predictive AI could also be used to improve the benefits and reduce the negative impacts of interventions.

Explainability and validation are crucial frontiers in predictive modeling. Although AI models are highly accurate, they possess a black-box nature-a feature that brings about concerns regarding trust and their use in clinical practice [41]. New developments in interpretable machine learning, however, are assisting in identifying the particular features—either morphological or molecular—that contribute to predictions. This does not only instill confidence in AI-driven insights but also introduces new hypotheses to be tested through experimental procedures.

## 8. Challenges and Limitations

Despite the remarkable progress in AI-driven histopathology for stem cell research, several challenges persist that limit its widespread adoption and translational impact. These challenges primarily revolve around data quality, variability in experimental methods, and unresolved ethical considerations that must be addressed to ensure reliability, reproducibility, and public trust.

### 8.1. Data Quality and Variability

The quality and heterogeneity of datasets are a vital limitation of the current AI-based histopathology. The majority of histopathology datasets are either small in size, restricted to a particular type of stem cells, or biased toward a particular mode of imaging [10]. The unwanted variability due to batch effects brought about by differences in tissue preparation, staining procedures, or microscope calibration can mask biological signals. For instance, H&E-stained slides cannot be cross-trained with AI training across different laboratories [24]. Furthermore, noise in training data is also caused by inconsistencies in annotation, which is usually performed by different pathologists with differing levels of expertise.

This is compounded by the fact that there are no standardized protocols in the preparation of tissues or in digital pathology pipelines. Unless they are harmonized, AI models that are trained in one lab might not perform successfully in a different lab [26]. In this regard, there is a need for large-scale, annotated, and standardized datasets that are curated by various institutions. Federated learning models and effective data augmentation plans can provide a viable solution to site-specific bias to allow AI pipelines to be generalizable to different histopathological conditions [135].

### 8.2. Ethical Considerations in AI Research

Besides technical issues, ethical issues are one of the most significant obstacles. Data privacy and informed consent are especially crucial in stem cell studies, where iPSCs or ESCs produced by patients are employed. Sharing histological images and related omics data with other institutions results in re-identification risks, despite the use of de-identified data [30].

Another challenge is the application of ESCs because, when AI is used alongside such studies, controversies arise regarding the moral use of embryos. Moreover, training datasets can introduce biases and unconsciously contribute to disparities in treatment results [23]. For instance, when AI models are unfairly trained on a particular ethnic or demographic population, they may not be generalizable across populations, which brings fairness and inclusivity issues [66]. Thus, ethical principles should be addressed together with AI devices to protect the privacy of data, enhance fairness in representation, and guarantee the reasonable use of sensitive stem cell data.

## 9. Future Directions

AI-enabled histopathology is moving from task-specific image analysis toward models that can combine visual, molecular, and textual information. Future development will depend on improving interpretability, generalizability, and collaborative learning and on the ability to integrate heterogeneous biological data while maintaining expert oversight [31,32,33].

### 9.1. Advancements in AI Technology

XAI remains important in understanding which morphological features influence model predictions and identifying failure patterns during validation. At the same time, pathology foundation models and visual language systems are expanding the range of tasks that can be addressed via shared pre-trained representations, including classification, retrieval, image-text interaction, and multimodal reasoning [31,32,33,41].

Federated learning provides a route for collaborative model development across institutions without centralizing raw whole-slide images, which may be useful when privacy and data governance requirements restrict direct data sharing [135]. Agentic AI represents another emerging direction, with recent systems using sequential evidence acquisition, adaptive slide navigation, and multistep reasoning to support pathological analysis [34,35]. Both approaches require multicenter validation and careful human oversight before wider translational use [34,35,135].

### 9.2. Integrative Approaches in Stem Cell Research

Future stem cell studies are expected to make greater use of multimodal models that combine morphology with genomic, transcriptomic, proteomic, functional, and clinical information. Spatial transcriptomics is especially relevant because it preserves the relationship between molecular signals and tissue architectures, while single-cell foundation models provide complementary representations of molecular cell states [37,62,115].

Longitudinal imaging and organoid models also provide opportunities to develop predictive systems that follow cellular changes over time and identify early features associated with later differentiation outcomes. For translational use, these models will need external validation across laboratories, transparent uncertainty assessment, and confirmation against molecular or functional endpoints so that computational predictions remain biologically interpretable and reproducible [29,60,135].

## 10. Case Studies

Real-world applications of artificial intelligence in stem cell histopathology demonstrate its capacity to transform both research and clinical practice. By highlighting successful implementations and comparing AI-driven methods with traditional pathology approaches, these case studies provide critical insights into the strengths, limitations, and future potential of computational approaches in regenerative medicine.

### 10.1. Successful Implementations of AI in Histopathology

Quantitative studies demonstrate the value of image-based AI for stem cell phenotyping. In human pluripotent stem cell cultures, machine learning models using cellular morphology alone achieved average phenotype prediction accuracy of 67%, while colony-level morphology reached 75%. Combining cellular and colony-level features increased the average accuracy to 99% with a random forest model and produced a receiver operating characteristic area under the curve of 0.997 [54].

DL has also been applied to the non-invasive quality control of human ESC colonies. A convolutional neural network classified colonies with favorable or unfavorable morphological phenotypes with 89% accuracy, and an image scale of approximately 144 μm provided the most informative spatial context for classification [55].

Recent work in retinal organoids extends this concept from classification to developmental prediction. A time-lapse dataset containing approximately 1000 organoids and more than 100,000 images was used to train deep learning models that predicted retinal pigmented epithelium formation with an F1 score above 0.85 at a median of 11 h after imaging began; the prediction of the final organoid morphology yielded an F1 score exceeding 70% at approximately 20 h [60].

### 10.2. Comparative Analysis of Traditional vs. AI Methods

Traditional histopathology remains important because expert interpretation provides a biological and contextual assessment of the tissue morphology [7]. Its performance can be affected by the workload, subjective interpretation, and inter-observer variability, particularly when large image collections or subtle phenotypic transitions are evaluated [3].

Quantitative comparisons show where computational analysis can add value. In PSC phenotype recognition, combined cellular and colony-level morphology yielded 99 ± 2% accuracy with random forest classification and 98 ± 2% with ANNs, compared with 67 ± 4% for cellular morphology alone using ANNs [54]. In retinal organoids, DL predictions of retinal pigmented epithelium formation reached an F1 score close to 0.9 across much of the imaging period and exceeded human prediction at the evaluated early time points [60].

These findings support a complementary workflow in which computational models provide scalable quantitative screening and experts provide the biological interpretation, verification, and assessment of uncertain or safety-relevant findings [54,55,60].

## 11. Collaboration Between Disciplines

Interdisciplinary collaboration is one of the key aspects in exploiting the full potential of AI in stem cell histopathology. The application of AI to biomedical research is neither a purely computational nor a purely biological issue, because it requires the combination of several fields that bring different experiences [83]. Through collaboration among the fields of pathology, computational science, molecular biology, and clinical medicine, the field will be able to create AI solutions that are scientifically sound and yet have clinical implications. This type of cooperation renders the whole research process-including data generation and analysis, the validation of the model, and its implementation in therapeutic use-more robust.

### 11.1. Interdisciplinary Research Teams

To overcome the lack of expertise that tends to slow down AI-driven initiatives, the creation of interdisciplinary research teams is significant. Pathologists offer invaluable experience with regard to the morphology of tissues and diagnostic criteria, whereas computational scientists can offer expertise in developing algorithms, machine learning, and the analysis of big data [82]. Molecular biologists provide insight into experiments and the functioning of cells, whereas clinicians can ensure that models are aligned with the needs of patients and the therapeutic realities. This is where cross-disciplinary training programs can be of use. These programs create a common language that can improve co-operation, training biologists to be computationally literate and data scientists to have biological literacy [82]. This synergy will facilitate more powerful experimental designs, knowledgeable data interpretation, and rigorous model validation, where AI outputs are scientifically correct and clinically pertinent.

### 11.2. Cross-Field Innovations

Cross-field innovations are also facilitated when researchers from different areas cooperate. Most AI techniques that were initially designed in fields like radiology and oncology are currently being reconfigured for stem cell applications. For instance, features extracted through radiomics, which extracts subtle imaging biomarkers for cancer diagnosis, can be reused to measure morphological changes in stem cells. Tumor segmentation algorithms, which are popular in defining the limits of malignant tissue, can provide guidance for the automated identification of differentiated and undifferentiated populations of stem cells [38,131].

## 12. Regulatory and Compliance Issues

The recent use of AI in stem cell histopathology creates regulatory and compliance issues that must be tackled urgently prior to the utilization of new tools in order to ensure significant clinical acceptance [136]. Although the potential offered by AI is scientifically promising, its safe and ethical use requires frameworks that can guarantee transparency, repetition, and trust by the public. Thus, ethical standards and regulatory bodies are the key influencing forces behind the future of AI-based healthcare innovations.

### 12.1. Regulatory Frameworks for AI in Healthcare

With the advancement of AI tools outside the research setting and into clinical practice, it is necessary to address regulation through relevant agencies, including the U.S. Food and Drug Administration (FDA), the European Medicines Agency (EMA), and other organizations located globally. Such agencies are becoming more concerned with the approval of AI-diagnostic and decision support systems [137,138]. In contrast to traditional medical devices, AI tools are dynamic; they can change as they are exposed to new data, thus creating regulatory challenges. In response, regulators are investigating adaptive frameworks that focus on the continuous monitoring of the performance of AI, as opposed to pre-approval validation.

Among these concerns is the ability of AI models to be generalized across different populations, laboratory facilities, and imaging circumstances. Models that work effectively in one situation but fail in another compromise both safety and trust. There has been a shift in regulatory guidelines regarding the need to prove robustness by utilizing multi-institutional datasets and independent validation cohorts [65]. Moreover, a record of the algorithmic decision-making process, risk management, and human control mechanisms is becoming a standard requirement. The end goal is to achieve innovation and patient safety while ensuring that AI systems yield reliable benefits without unintended harm.

### 12.2. Compliance with Ethical Standards

Regulatory control should not solely be confined to technical validation but include other standards of ethical conduct. Transparency, accountability, and fairness are of particular concern in relation to stem cell research, where tissues taken from patients and sensitive genetic data are often utilized. The design of AI models should be conducted with specific consideration of informed consent, which requires that the patient is informed of how his or her biological information can be utilized in the training of the algorithm and in subsequent applications.

Fair representation in training data is another ethical consideration. If AI systems are trained disproportionately on data provided by certain groups of people, there is a high risk that they will incorporate biases that will undermine the safety and effectiveness of regenerative therapeutic interventions in different populations. Active mitigation measures to reduce bias, such as balanced data collection, fairness-sensitive algorithms, and regular auditing, are thus needed. Meanwhile, measures of accountability should be developed to define accountability in the event that AI-assisted decisions produce negative results.

By combining strong regulatory guidelines with stringent ethical protection, it will be possible to advance AI-enhanced histopathology in a responsible manner. This will not only ensure that clinical translation becomes easier but will also enhance the level of public trust, so that the potential transformative value of AI in stem cell medicine can be exploited in a safe and just manner.

## 13. Conclusions

AI is transforming histopathology into a data-driven science with the ability to not only describe but also predict and explain cellular processes in stem cell research. AI has provided the first-ever opportunity to harmonize structural and molecular views by integrating the morphology of the cell, functional assays, and multi-omics analysis. Histopathology in the traditional form was highly dependent on the interpretation of experts; this method, despite its invaluability, was subjective and affected by inter-observer variability, as well as not being applicable to large datasets. Conversely, AI-based tools, especially those utilizing machine learning and deep learning, can analyze thousands of histological images with both higher speeds and greater accuracy than humans. This enables researchers to reveal minute morphological aspects associated with stem cell conditions and to relate them to genomic and proteomic indicators, contributing to our knowledge of stem cell fates and functionality.

The uses of AI in stem cell histopathology include the automated identification of stem cell subtypes and the prediction of stem cell differentiation and therapeutic responses. Automated classifiers have transformed previously labor-intensive analyses to ensure fast quality control in regenerative medicine workflows and drug discovery pipelines. Meanwhile, predictive models combine longitudinal imaging with multi-omics data to predict cell behavior, providing clinicians and researchers with practical information regarding safety, efficacy, and even patient-specific responses. These developments are already benefiting regenerative medicine, enabling personalized therapies, and creating new possibilities in the field of tailored treatment plans.

This transformational potential is not without major challenges. The quality and variability of data remain a limitation to generalizability, with batch effects caused by differences in tissue preparation, staining, and imaging protocols potentially biasing AI models. Moreover, the ethical aspects of AI-stem cell studies, including privacy and consent involved in data sharing or inequity and fairness in model predictions, should be addressed to guarantee public confidence and ethical use. The unification of histopathological processes, pipeline creation, and increased openness in AI decision-making are thus crucial measures.

In the future, with innovative designs, the industry will be taken to a new level. With XAI, researchers and clinicians will demonstrate increased trust because computational predictions will become transparent and interpretable. Federated learning offers solutions to collaborative training on various datasets without violating patient privacy. Meanwhile, multimodal models based on the combination of spatial transcriptomics, imaging, and clinical data will serve as comprehensive models of stem cell biology and therapy. All of these developments will result in a paradigm shift in which AI will assist in supporting-and may even redefine the role of-histopathology in translational medicine, eventually facilitating a transition between scientific discovery and clinical practice.

## 14. Recommendations

The achievements of AI integration in stem cell histopathology need to be consciously oriented toward the need to fill existing gaps in data, experience, regulation, and translation. One of the main suggestions is investment in annotated, high-quality datasets. Currently, inconsistencies in staining procedures, imaging systems, and annotation habits negatively affect the applicability of AI models. Researchers can create AI systems that work consistently and in different experimental and clinical contexts by creating large, standardized repositories that are curated institute-wide and with strong quality control. Federated learning and collaborative consortia models can also increase the diversity of datasets and preserve the privacy of patients, which will result in representative and resilient AI models.

The other priority is to promote interdisciplinary training. The sophistication of AI–stem cell integration requires the skills of computational science, pathology, stem cell biology, and clinical medicine. The systems of the past cannot be used to meet the challenges of today regarding data integration, model development, and clinical validation, which are multifaceted and necessitate new solutions. The first priority should be interdisciplinary training programs, as well as dual-degree opportunities and cross-laboratory fellowships, seeking to develop researchers who are knowledgeable not only in the field of computational science but also in biomedical science. These efforts will promote cooperation, decrease communication barriers, and speed up translational impacts.

It is also necessary to develop effective regulatory frameworks. The FDA and EMA are starting to develop instructions for AI-related diagnostics, but there are not yet clear guidelines that are adapted to the specifics of stem cell research. Rules should also be transparent and reproducible and enable the post-deployment monitoring of AI systems but must also be flexible to changes in technology. It will be essential to harmonize standards worldwide, since the development of both stem cell research and AI is global.

Explainability and fairness should be encouraged in order to build trust. XAI techniques are capable of producing clear outputs to aid researchers and clinicians in comprehending model decisions so as to overcome the doubts linked to black-box systems. Simultaneously, it is necessary to promote fairness by using balanced datasets and applying bias reduction measures in order to eliminate inequities in therapeutic outcomes among different populations.

Lastly, there should be an attempt to fortify translational pipelines. Innovation encompassing both AI and stem cell concepts in the laboratory and clinic must be ensured through a continuous series of pre-clinical validation, regulatory approval, and clinical validation. Stronger partnerships among academia, industry, and healthcare providers will also help to guarantee that AI-driven insights are not restricted to the research environment but converted into real patient impacts. The potential of AI in regenerative medicine can be exploited by strengthening these pipelines.

## Data Availability

No new data were created or analyzed in this study. Data sharing is not applicable to this article.
